# Case Series: Extracorporeal Shockwave Myocardial Revascularization Therapy Improves Ischemic Response, Functional Capacity, and Quality of Life in Indicated CABG-Stable Angina Pectoris Patients

**DOI:** 10.3389/fcvm.2022.799834

**Published:** 2022-02-11

**Authors:** Mohammad Rizki Akbar, Dwi Laksono Adiputro, Badai Bhatara Tiksnadi, Erwin Affandi Soeriadi, Melawati Hasan, Fauzan Muttaqien, Pradana Pratomo Raharjo, Eliza Nurazizah, Najmi Fauzan Tarsidin

**Affiliations:** ^1^Department of Cardiology and Vascular Medicine, Faculty of Medicine, Dr. Hasan Sadikin General Hospital, Padjadjaran University, Bandung, Indonesia; ^2^Department of Cardiology and Vascular Medicine, Faculty of Medicine, Ulin General Hospital, Lambung Mangkurat University, Banjarmasin, Indonesia; ^3^Department of Nuclear Medicine and Molecular Imaging, Faculty of Medicine, Dr. Hasan Sadikin General Hospital, Padjadjaran University, Bandung, Indonesia

**Keywords:** cardiac shock wave, case series, functional capacity, stable angina, quality of life

## Abstract

**Introduction:**

Extracorporeal shockwave myocardial revascularization (ESMR) is included in the guidelines only for patients with refractory angina pectoris having no option for invasive revascularization. We intend to report a case series with ESMR therapy is indicated patients with coronary artery bypass grafting-stable angina pectoris (CABG-SAP) who refuse the surgery, irrespective of angina symptoms.

**Methods:**

We review medical records of patients with SAP admitted to ESMR therapy in Dr. Hasan Sadikin General Hospital, Bandung, Indonesia from January 2018 to December 2019. Recorded variables at baseline and after therapy extracted, namely, (1) ischemic response, double product, and (2) functional capacity measured as metabolic equivalent (MET) using treadmill test; (3) six-minute walking test distance achieved; and (4) quality of life using SF-36 Questionnaire.

**Results:**

A total of four indicated patients with CABG-SAP from 50 to 75 years old were included in this study. At baseline, one patient is CCS class I and two patients are CCS class II with SDS ranging from 3 to 17. Ischemic response improved in all the patients. The double product improved in patient 1 9,600–14,872 mm Hg × bpm, patient 2 9,460–10,640 mm Hg × bpm, and patient 4 17,220–20,480 mm Hg × bpm. The functional capacity improved in Patient 1 8.07–8.91 METs, patient 2 1.91–4.01 METs, patient 3 3.45–6.39 METs, and patient 4 3.9–4.43 METs. The 6-min walking distance improved in patient 1 540–570 m and patient 2 345–405 m. The CCS class, bodily pain, and general health domain scores improved in all patients.

**Conclusion:**

ESMR therapy might be beneficial for indicated patients with CABG-SAP to improve ischemic response, functional capacity, and physical component of quality of life.

## Introduction

Coronary artery disease (CAD), including stable angina pectoris (SAP), remains the leading cause of mortality and morbidity in Indonesian adults (1–3). The current SAP treatment guideline mainly focuses on medical therapy and invasive revascularization approaches (1). Studies showed that invasive revascularization therapy leads to better anginal pain reduction and improved quality of life than using medication alone (4). However, approximately, one-third of patients with CAD in Indonesia refuse invasive treatment approaches (5).

The mechanism that precipitates angina in patients with SAP highly correlates with the multifaceted mechanism of chronic myocardial ischemia (6). Imbalances of oxygen supply-demand, anaerobic metabolism, sympathetic activation, and myocardial perfusion have been postulated to influence myocardial ischemia (7). In addition, coronary collaterals could also affect myocardial perfusion, improving myocardial ischemia (8). Exercise and some emerging non-invasive treatments have been studied to improve microvascular circulation in patients with CAD (9–11).

Extracorporeal shockwave myocardial revascularization (ESMR) is a non-invasive procedure that has a beneficial effect in patients with SAP having no option for invasive revascularization and refractory angina (12). In a triple-blind randomized clinical trial of patients with refractory angina, ESMR therapy demonstrated significantly improved myocardial perfusion during 6-month follow-up compared to medical therapy alone (9). A recent meta-analysis comparing ESMR vs. optimal medical therapy alone in patients with refractory angina shows a significant improvement in the 6-min walking test distance achieved and quality of life measured using the Seattle angina questionnaire (13). A limited study describes the efficacy of ESMR to treat indicated invasive revascularization patients with SAP. We report our case series of indicated patients with CABG-SAP, irrespective of angina status undergoing ESMR therapy.

## Methods

We conducted a retrospective case series study of prospectively collected data of patients who have completed ESMR therapy in the Department of Cardiology and Vascular Medicine, Dr Hasan Sadikin General Hospital, Bandung, Indonesia, between January 2018 and December 2019. The inclusion criteria were patients with SAP who completed nine ESMR therapy sessions and indicated a CABG procedure after angiography. The exclusion criteria were a history of invasive revascularization. We collected data from medical records concerning clinical characteristics, ESMR therapy protocol, baseline evaluation, and postintervention evaluation. In this report, we focus to report data of 6-min walking distance, treadmill test, and quality of life measured using the Canadian Cardiology Society Class (CCS Class) and the Short Form-36 (SF-36) questionnaire before and after ESMR therapy. Another report for perfusion and contractility changes is published elsewhere (14). The study was approved by the Health Research Ethics Committee of Dr. Hasan Sadikin General Hospital Bandung (No. LB.02.01/X.6.5.284/2020) and signed individual consent for this report was waived.

## Results

We included records of four patients with SAP having Syntax scores ranging from 32 to 43. All the patients are suggested for CABG procedure by their interventional cardiologist. However, the patient refuses to surgery approach and they undergo ESMR therapy in our center based on clinician referral. Clinical and demographic features are shown in Tables 1, 2. The mean age was 60 years old (range 50–75 years old) consisted of three males and one female. Patients 1, 3, and 4 were diagnosed with the three-vessels disease (3VD), and Patient 2 was diagnosed with 3VD with left-main involvement. Patient 2 has a history of myocardial infarction. The mean left ventricular ejection fraction was 62.5 ± 3.69%. The mean of Summed Stress Score (SSS), Summed Difference Score (SDS), and Summed Rest Score (SRS) were 17.25 ± 9.8, 9.5 ± 5.9, and 7.75 ± 5.5, respectively. Patients 1, 2, and 4 have hypertension as risk factors, Patient 1 also has psychological stress as risk factors, and Patient 4 has smoking and diabetes as risk factors. All four patients have no history of invasive revascularization and refused the CABG procedure. All four patients completed ESMR therapy in addition to their optimal medical therapy.

**Table 1:** Included patients characteristics.

**Variable**	**Patient 1**	**Patient 2**	**Patient 3**	**Patient 4**
Gender	Male	Male	Male	Female
Age (years)	54	61	75	50
BMI (kg/m^2^)	26.6	25.3	24.6	24.6
Blood pressure (mmHg)	90/70	120/70	140/90	140/90
Heart rate (bpm)	53	63	56	81
CAD history (years)	10	2	2	2
**Coronary stenosis**				
LAD	Yes	Yes	Yes	Yes
LCX	Yes	Yes	Yes	Yes
RCA	Yes	Yes	Yes	Yes
LM	No	Yes	No	No
SYNTAX score	41	32	43	40
History of myocardial infarct	No	Yes	No	No
Biplane simpson LVEF (%)	62	63	58	67
**SPECT**				
Areas with reduced blood infusion	Left ventricular apex, apical lateral wall, and middle until basal inferolateral wall	Left ventricular apex, apical anterior wall, apical lateral wall, and middle inferolateral wall	Left ventricular middle until basal inferior wall, and anteroseptal apex	Left ventricular apical anteroseptal wall, basal anteroseptal wall
SSS	31	13	17	8
SDS	17	3	11	7
SRS	14	10	6	1
**Risk factor**				
**a. Non-modifiable**				
- History of premature CAD in first-degree relatives	No	No	No	Yes
**b. Modifiable**				
- Obesity	Yes	No	No	No
- Hypertension	Yes	Yes	No	Yes
- Hypercholesterolemia	No	No	Yes	No
- Diabetes Mellitus	No	No	No	Yes
- Smoking	No	Yes	No	Yes
- Stress	Yes	No	No	No

*BMI, body mass index; CAD, coronary artery disease; CCS, Canadian Cardiovascular Society Class; LAD, left anterior descending artery; LCx, left circumflex artery; LM, left main artery; LVEF, left ventricular ejection fraction; RCA, right coronary artery; SYNTAX, Synergy between Percutaneous Coronary Intervention with TAXUS and Cardiac Surgery*.

**Table 2:** Medications history of included patients.

	**Before ESMR**	**After (3-month) ESMR**
Patient 1	Clopidogrel 1 x 75 mg	Clopidogrel 1 x 75 mg
	Bisoprolol 1 x 10 mg	Bisoprolol 1 x 5 mg
	Amlodipine 1 x 5 mg	Amlodipine 1 x 5 mg
	Candesartan 1 x 16 mg	Candesartan 1 x 16 mg
	Glyseril-tri-nitrate 2 x 2.5 mg	Glyseril-tri-nitrate 2 x 2.5 mg
	Atorvastatin 1 x 40 mg	Trimetazidine 2 x 35 mg
		Atorvastatin 1 x 40 mg
Patient 2	Aspirin 1 x 80 mg	Aspirin 1 x 80 mg
	Bisoprolol 1 x 10 mg	Clopidogrel 1 x 75 mg
	Amlodipine 1 x 10 mg	Bisoprolol 1 x 7.5 mg
	Candesartan 1 x 16 mg	Amlodipine 1 x 10 mg
	Glyseril-tri-nitrate 2 x 2.5 mg	Candesartan 1 x 4 mg
	Hydrochlorothiazide1 x 25 mg	Glyseril-tri-nitrate 2 x 2.5 mg
	Atorvastatin 1 x 20 mg	Atorvastatin 1 x 20 mg
Patient 3	Aspirin 1 x 80 mg	Aspirin 1 x 80 mg
	Bisoprolol 1 x 10 mg	Bisoprolol 1 x 10 mg
	Glyseril-tri-nitrate 2 x 2.5 mg	Glyseril-tri-nitrate 2 x 2.5 mg
	Atorvastatin 1 x 40 mg	Furosemide 1 x 20 mg
		Atorvastatin 1 x 40 mg
Patient 4	Clopidogrel 1 x 75 mg	Clopidogrel 1 x 75 mg
	Bisoprolol 1 x 10 mg	Bisoprolol 1 x 10 mg
	Amlodipine 1 x 10 mg	Amlodipine 1 x 10 mg
	Candesartan 1 x 16 mg	Candesartan 1 x 8 mg
	Glyseril-tri-nitrate 2 x 2.5 mg	Glyseril-tri-nitrate 2 x 2.5 mg
	HCT 1 x 12.5 mg	Trimetazidine 2 x 35 mg
	Atorvastatin 1 x 40 mg	Atorvastatin 1 x 40 mg

This therapy consisted of nine sessions for 3 months with three sessions per week and was performed on the 1st, 5th, and 9th intervention weeks. During the 1st, 5th, and 9th intervention weeks, the shockwave was delivered to the target spots segments of the left ventricle, respectively, for 30 min each session. A 3-week therapy-free interval was kept after the 1st and 5th therapy weeks (15).

**Figure 1:**
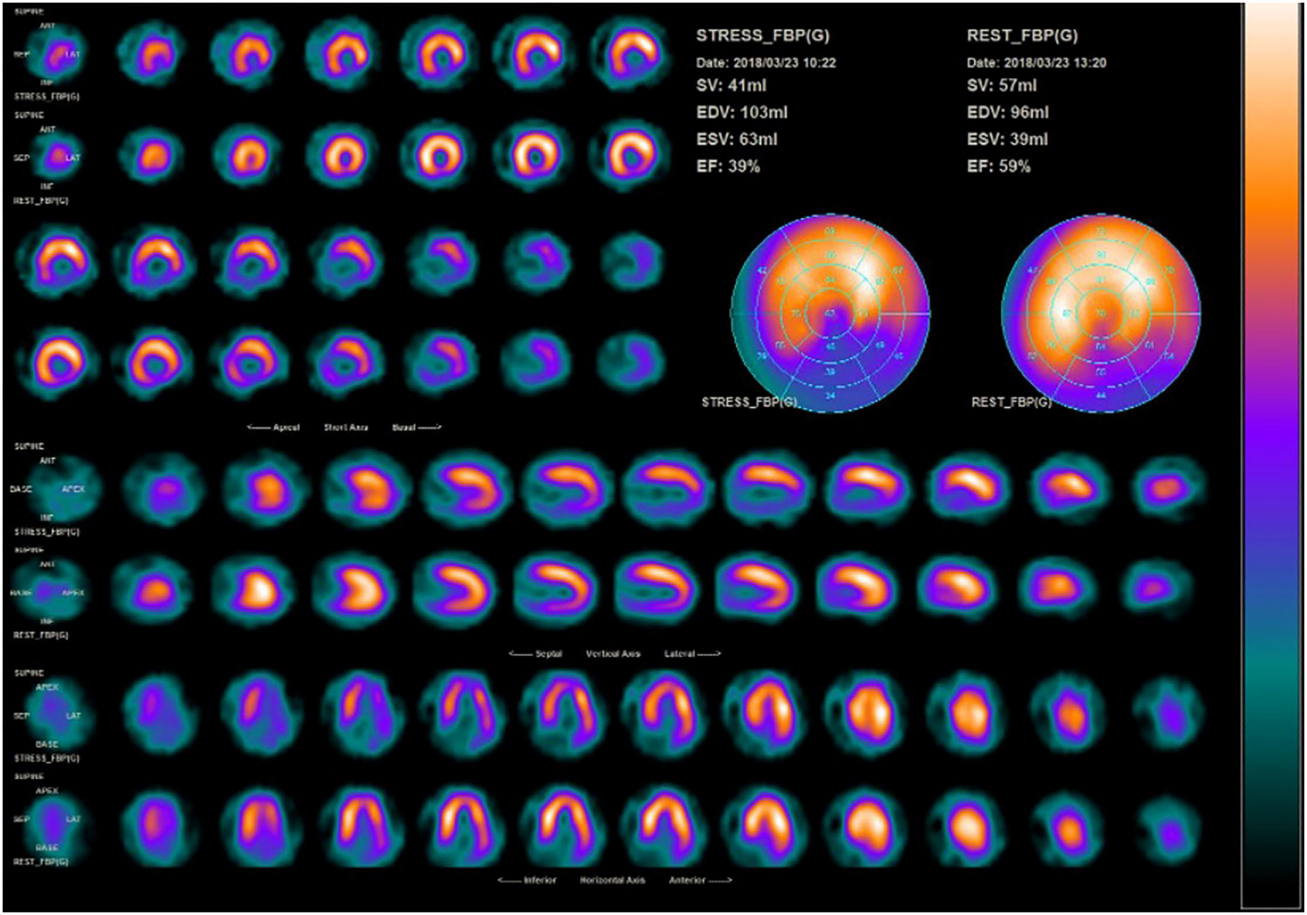
SPECT examination of patient 1 before ESMR therapy.

At first, shockwave (SW) is delivered to the border zone to induce neovascularization from the segment with adequate blood supply to the ischemic zone. The ischemic zone was divided into specific target spots based on one focus zone of the SW applicator to achieve optimal therapy. The SW applicator was fixed at the measured distance when end-diastole. An inflatable silicone cushion was filled, and the ultrasound gel was used for optimal delivery of shockwaves into the body. Low-intensity SW (100 impulses/spot) were delivered to the target spot under electrocardiographic R-wave gating. The target spot is different for each individual, and if there were five target spots, the total SW are 500 impulses (15). The target spot is determined using the single-photon emission CT (SPECT) test and defined as areas with reduced blood infusion. An example of SPECT examination report represented by Patient 1 is given in Figure 1.

**Figure 2:**
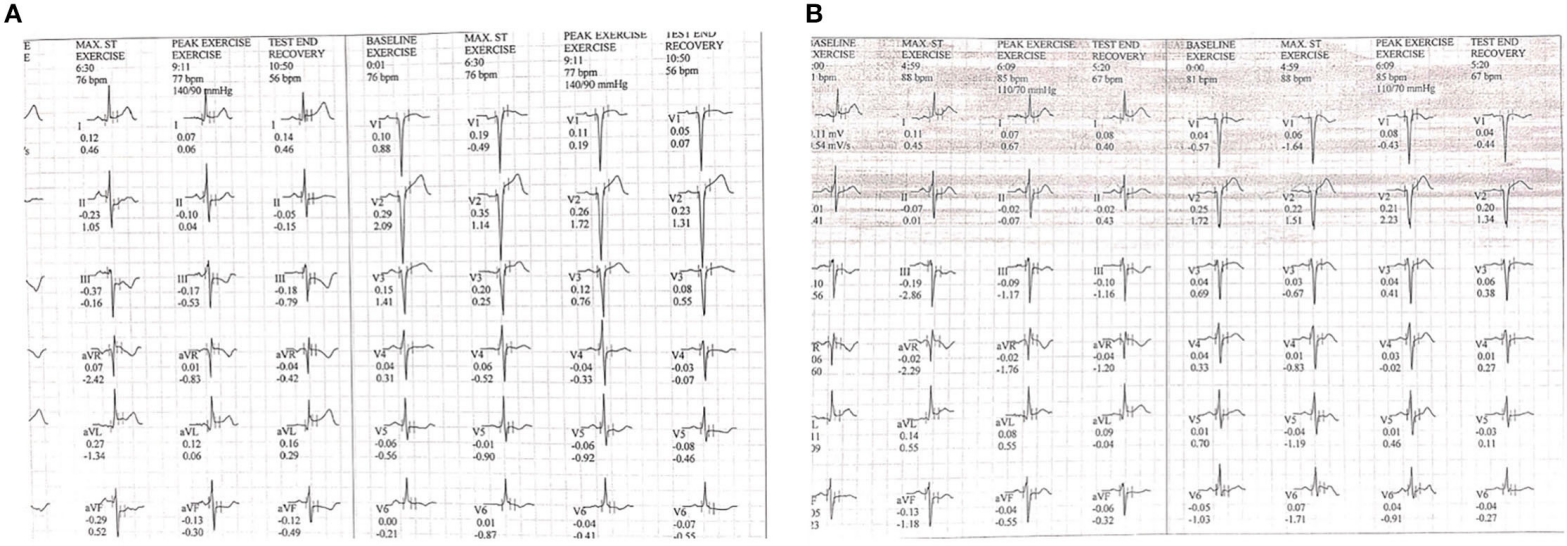
ECG changes during treadmill test at **(A)** baseline and **(B)** after ESMR therapy.

During 3 months after ESMR therapy, post-ESMR evaluations were collected. Therapy efficacy in this study is presented in Table 3. Patient 2 experienced limiting angina at baseline during the treadmill stress test that improved after ESMR therapy. The ischemic response is improved in patient 1 positive to negative, patient 2 suggestive to negative (Figure 2), and patients 3 and 4 positive to suggestive ischemic responses. The double product (DP) improved in patient 1 9,600–14,872 mm Hg × bpm, patient 2 9,460–10,640 mm Hg × bpm, and patient 4 17,220–20,480 mm Hg × bpm. The functional capacity improved in patient 1 8.07–8.91 METs, patient 2 1.91–4.01 METs, patient 3 3.45–6.39 METs, and patient 4 3.9–4.43 METs. The 6-min walking distance improved in patient 1 540–570 m and patient 2 345–405 m, but deteriorated in patient 3 435–405 m and patient 4 450–425 m.

**Table 3:** The effect of ESMR therapy to ischemic response, functional capacity, and quality of life.

	**Patient 1**		**Patient 2**		**Patient 3**		**Patient 4**	
	**Before**	**After**	**Before**	**After**	**Before**	**After**	**Before**	**After**
**Functional capacity**								
**Treadmill stress test**								
Chest pain during test	+ at recovery	+ limiting angina	+ limiting angina	-	-	-	-	-
Ischemic response	+	-	±	-	+	±	+	±
METs	8.07	8.91	1.91	4.01	3.45	6.39	3.59	4.43
DP (mmHg*bpm)	9,600	14,872	9,460	10,640	10,920	10,890	17,220	20,480
6MWD (m)	540	570	345	405	435	405	450	425
**Quality of life**								
Angina	+	+	+	+	+	+	+	-
CCS class	III	I	II	I	I	I	I	-
**SF-36**								
**Physical health**								
FP	50	80	65	70	70	50	55	35
RP	0	0	0	75	0	0	0	50
BP	68	90	58	80	65	78	58	68
GH	65	85	40	65	70	75	65	75
**Mental health**								
RE	0	0	0	100	33	0	0	33
VT	60	20	30	55	45	75	65	45
MH	84	60	40	64	76	80	84	72
SF	63	75	63	88	75	62	100	63

*+, positive ischemic response; -, negative ischemic response; ±, suggestive ischemic response; 6MWD, 6-min walking distance; BP, bodily pain; CCS Class, Cardiovascular Canadian Society Class; FP, physical function; GH, general health; METs, metabolic equivalents; MH, mental health; RE, role limitations due to emotional health; RP, role limitations due to physical health; SF, social function; VT, vitality*.

After ESMR, there are improvements of CCS Class in patient 1 CCS III to I and patient 2 CCS II to I. In addition, our case series demonstrated the various effect of ESMR therapy on quality of life measured using SF-36. The bodily pain domain score improved in all the patients as seen in patient 1 60–90, patient 2 58–80, patient 3 65–78, and patient 4 58–68. The general health domain score improved in all the patients as seen in patient 1 65–85, patient 2 40–65, patient 3 70–75, and patient 4 65–75. On medical records, there are reductions of ß-blocker dose in patients 1 and 2. Also, there are additions for antianginal medication such as trimetazidine in patients 1 and 4. During a 3-month follow-up period, no major adverse cardiovascular events (MACEs) such as death, myocardial infarction, coronary revascularization, stroke, and hospitalization because of heart failure were recorded in all the patients.

## Discussion

Based on the current guidelines, the position of ESMR therapy is recognized as a potential treatment for refractory angina (1). Previous clinical studies of ESMR found beneficial effects for patients with refractory angina pectoris that do not have options for invasive approaches (13). However, most EMSR therapy trials only include a small sample of patients for each group (16–18). Trials with large samples are needed to increase the power of this therapy.

This case series demonstrated its novelty by evaluating the use of ESMR therapy in patients with SAP currently indicated to CABG procedure, irrespective of angina symptoms. Therefore, the included patients are not yet defined as patients with refractory angina pectoris. In our study, ESMR therapy could improve ischemic response, functional capacity, angina symptoms, and physical component quality of life, especially in bodily pain and the general health domain.

Extracorporeal shockwave myocardial revascularization (ESMR) therapy works by triggering the inertial collapse of micro-sized bubbles, which exert shear stress-like forces on myocardial tissue. Therefore, stimulating angiogenesis induction of growth factors, namely, vascular endothelial growth factor (VEGF) and nitric oxide synthase (NOS) (19). In animal studies, increased VEGF and NOS concentration after ESMR therapy was also observed. These substances stimulate the process that increases collateral growth, myocardial remodeling, and left ventricular compliance (20). In previously published trials, ESMR therapy was demonstrated to improve myocardial perfusion, clinical symptoms, and functional capacity in patients with refractory angina pectoris (16–18).

The mechanism that induces angina might differ based on coronary conditions in patients with refractory angina and SAP that still indicated invasive revascularization. In patients with refractory angina, anginal pain is mainly due to other factors beyond significant large coronary stenosis, such as microvascular coronary disorders and vasospasm (12). Whereas, in indicated patients with CABG-SAP, significant epicardial stenosis is present, causing a significant mismatch of oxygen demand in the myocardium, provoking angina and reduced quality of life (21).

As demonstrated in this case series, ESMR therapy could improve ischemic response and functional capacity in patients with remaining significant stenosis in epicardial coronary circulations. It may be postulated that ESMR, with its mechanism, could improve myocardial microcirculation and coronary collaterals (9). This improvement leads to improved myocardial ischemia response and improves functional capacity and physical component of quality of life. Therefore, our small case series might broaden the clinical study of ESMR therapy to this population than its previous use in refractory angina.

In our patients, Ischemic response improved as shown in the double product, ischemic electrocardiogram (ECG) changes findings and maximal functional capacity during treadmill stress test after ESMR therapy. Trial of ESMR therapy in patients with refractory angina pectoris by Shkolnik et al. (22) comparing 35 patients undergoing ESMR with 37 patients undergoing placebo demonstrated improvement of exercise duration during treadmill test with the mean increase in the exercise duration was 32.8 ± 83.3 s at 3-months and 48.8 ± 106.5 s at the 6-months follow-up in the ESMR therapy group compared with 68.3 ± 119.5 and 80.4 ± 128.8 s in the placebo group. Although exercise duration in ESMR groups does not show better than placebo in that trial, the number of patients with ischemic response recorded during peak exercise decreased significantly to only 18 patients in the ESMR group compared with 33 patients in the placebo group at 6-month follow-up. These findings mean that non-cardiac reasons may limit exercise duration in the included patients. In addition, in our findings, ESMR improves myocardial oxygen consumption, as shown in the improvement of double product. Therefore, an improvement in the double product could reflect an improvement of exercise tolerance that correlates with myocardial activity (23).

In the context of 6-min walking distance evaluation after ESMR therapy in patients with refractory angina pectoris, a trial by Weijing et al. (24) comparing 46 patients in ESMR therapy with 41 patients receives medical therapy. That study demonstrated an improvement of 6-min walking distance from 331.7 ± 62.3 to 403.1 ± 61.2 m in the ESMR therapy group compared 319.3 ± 69.3 to 336.7 ± 71.1 m in the medical therapy group. However, our case series only demonstrate an improvement of the 6-min walking distance achieved only in Patients 1 and 2. The 6-min walk distance measures the maximum distance that a person can walk in 6 min. This test might be reflecting the capability of patients in normal daily activities and related to mobility ability (25). Although it has good reproducibility, the 6-min walk test is not without limitations. The test does not provide insight into the individual functional capacity. Its results can be affected by several unrelated independent factors, namely, age, height, weight, impaired cognition, and patient motivation. Therefore, these factors should be considered when interpreting the test results (26). Deterioration of the 6-min walk distance achieved after ESMR therapy in Patients 3 and 4 may be due to advanced age, diabetes, and smoking activity that can negate and shorten a 6-min walk distance (27, 28).

In this study, CCS Class improved in all the patients. In addition, we also measured quality of life using SF-36. This questionnaire contains parameters that can assess the physical and mental health of patients (29). We found that after ESMR therapy, Patient 2 has improved in physical and mental health domain quality of life. Improvement in ischemic response and functional capacity in Patient 2 leads to improvement in physical performance. In general, improvement in physical performance is linked to improvement in quality of life (30). The bodily pain and general health domain of physical health component quality of life scores improved in all patients in our case series after ESMR therapy. Concordance with our findings, a meta-analysis study found ESMR therapy could improve CCS class up to one severity class (13). Another study by Schmid et al. (18) demonstrated improvement of general health, physical function, and vitality domain in 11 patients with refractory angina after ESMR therapy.

This case study had some limitations. Since the data were derived retrospectively from medical records, several factors cannot be controlled such as reasons and strategies for medications modifications that could be affected post-therapy evaluation. In our report, there are changes in the dose of ß-blocker dose and the addition of anti-ischemic medications in some patients that might be affecting their exercise tolerability. Also, only four patients were assessed in this study, limiting clinical interpretations of this study. Last, due to the lack of a control group, our findings may result in high bias and cannot be justified as a clinical recommendation.

Our report will stand out its novelty that represents the effect of ESMR in patients with SAP that not defined as patients with refractory angina pectoris. This report will complement our previous report for positive changes in myocardial perfusion and contractility in the included patients (14). Therefore, our findings could broaden the target population for further clinical studies of ESMR therapy in various subsets of patients with SAP. Future trials using ESMR therapy that includes patients with SAP suitable for invasive revascularization are needed to confirm our findings. The study should be with control groups, and confirmatory myocardial function and perfusion evaluation are needed along with functional capacity assessment to match clinical improvement. We are concerned that ESMR therapy did not replace the position of the invasive approach to treating significant epicardial stenosis in patients with SAP.

## Conclusion

There are improvements of ischemic response, functional capacity, and physical component quality of life in four indicated patients with CABG-SAP who received ESMR therapy evaluated in this study. We are concerned that there are marked limitations in our study. Therefore, more rigorous studies with controlled trials and large samples are needed to confirm these findings.

## Data Availability Statement

The original contributions presented in the study are included in the article/supplementary material, further inquiries can be directed to the correspondingauthor/s.

## Ethics Statement

The studies involving human participants were reviewed and approved by Health Research Ethics Committee of Dr. Hasan Sadikin General Hospital Bandung. The patients/participants provided their written informed consent to participate in this study. Written informed consent was obtained from the individual(s) for the publication of any potentially identifiable images or data included in this article.

## Author Contributions

MA and ES: conception, design, supervision, materials, data collection and processing, analysis and interpretation, writing, and critical review. DA: conception, design, supervision, materials, funding, analysis and interpretation, and critical review. BT: conception, design, supervision, materials, data collection and processing, analysis and interpretation, literature review, writing, and critical review. MH: supervision, materials, data collection and processing, analysis and interpretation, writing, and critical review. FM: supervision, materials, funding, analysis and interpretation, and critical review. PR and EN: materials, data collection and processing, analysis and interpretation, writing, and critical review. NT: analysis and interpretation, literature review, writing, and critical review. All authors contributed to the article and approved the submitted version.

## Funding

This study was funded by the Ulin General Hospital, Banjarmasin, Indonesia.

## Conflict of Interest

The authors declare that the research was conducted in the absence of any commercial or financial relationships that could be construed as a potential conflict of interest.

## Publisher's Note

All claims expressed in this article are solely those of the authors and do not necessarily represent those of their affiliated organizations, or those of the publisher, the editors and the reviewers. Any product that may be evaluated in this article, or claim that may be made by its manufacturer, is not guaranteed or endorsed by the publisher.
